# GEROFIT TELEHEALTH-DELIVERED GROUP EXERCISE IMPACTS SOCIAL SUPPORT AND HEALTH AMONG OLDER VETERANS

**DOI:** 10.1093/geroni/igac059.1272

**Published:** 2022-12-20

**Authors:** Lauren Abbate, Rebekah Harris, Sarah Jordan, Kathryn Nearing, Janet Prvu Bettger, Megan Pearson, Miriam Morey, Katherine Hall

**Affiliations:** Eastern Colorado Health Care System, Aurora, Colorado, United States; VA Boston, Boston, Massachusetts, United States; University of Colorado, school of Medicine, Aurora, Colorado, United States; VA Eastern Colorado Geriatric Research Education and Clinical Center, Denver, Colorado, United States; Duke University, Durham, North Carolina, United States; Durham VA Healthcare System, Durham, North Carolina, United States; Durham VA Health Care System, Durham, North Carolina, United States; Durham VA Health Care System, Durham, North Carolina, United States

## Abstract

Group exercise provides social support, which is especially important among older adults. Gerofit conducted a telephone survey with participants (n=258) from 15 sites to learn about Veterans’ experiences with transition to Gerofit-specific telehealth-delivered group exercise to guide continuous improvement of the program. One, open-ended question was asked at the end, inviting respondents to reflect on any other thoughts they had about the telehealth-delivered exercise sessions. This yielded robust qualitative responses which constitute the data analyzed in this qualitative descriptive study. Key themes that emerged from participant responses included: 1) telehealth-delivered group exercise provides structure, motivation, and accountability; and 2) transitioning from in-person to telehealth-delivered exercise has many beneficial impacts on health such as retaining strength and ability to recover from injury, weight management, and positive impacts on mental health and quality of life. Telehealth-delivered group exercise sessions provide social support and are associated with positive health impacts.

